# X-ray diffraction and NMR data for the study of the location of idebenone and idebenol in model membranes

**DOI:** 10.1016/j.dib.2016.03.064

**Published:** 2016-03-24

**Authors:** Victoria Gómez-Murcia, Alejandro Torrecillas, Ana M. deGodos, Senena Corbalán-García, Juan C. Gómez-Fernández

**Affiliations:** Departamento de Bioquímica y Biología Molecular A, Universidad de Murcia, IMIB-Arrixaca, Campus of International Excellence “Campus Mare Nostrum”, Murcia, Spain

**Keywords:** Small angle X-ray diffraction, Wide angle X-ray diffraction, 31P NMR, 2H NMR

## Abstract

Here we present some of our data about the interaction of idebenone and idebenol with dipalmitoyl-phosphatidylcholine (DPPC). In particular, we include data of small angle X-ray diffraction (SAXD) and wide angle X-ray diffraction experiments, obtention of electronic profiles of the membranes, ^2^H-NMR and ^31^P-NMR, as part of the research article: “Both idebenone and idebenol are localized near the lipid-water interface of the membrane and increase its fluidity” (Gomez-Murcia et al., 2016) [1]. These data were obtained from model membranes that included different proportions of idebenone and idebenol, at temperatures both above and below of the gel to fluid phase. The X-ray experiments were carried out by using a modified Kratky compact camera (MBraun-Graz-Optical Systems, Graz Austria), incorporating two coupled linear position sensitive detectors. The NMR data were collected from a a Bruker Avance 600 instrument.

**Specifications Table**

**Table:** 

Subject area	Biology
More specific subject area	Biophysical Chemistry of membranes
Type of data	Table, figure
How data was acquired	X-ray diffraction; NMR
Data format	Raw and analyzed
Experimental factors	Samples prepared as described in Gómez-Murcia et al. [1].
Experimental features	SAXRD form DPPC-idebenol samples; *d*-spacings of samples DPPC/idebenone and DPPC/idebenol; electronic profiles of the membranes obtained from the diffractograms; ^31^P-NMR and ^2^H-NMR of d62-DPPC/idebenone and d_62_-DPPC/idebenol
Data source location	*Murcia, Spain*
Data accessibility	*Data is with this article*

**Value of the data**

• These diffractograms, electronic profiles and spectra may be useful to establish comparisons with the data obtained by other workers exploring these biomolecules or other similar.
• Providing these details to the scientific community may be of help when trying to interpret experiments carried out by using the same techniques.
• The supplied repeat distances obtained from X-ray diffraction may be of great help to study in detail these systems.

## 1 Data

These data were obtained as described in detail in [1]. SAXD diffractograms, SAXD and WAXD spacings, 31P NMR and 2H NMR are included obtained from idebenone-DPPC and idebenol/DPPC samples.

## 2 Experimental design, materials and methods

**Materials**. 1-Palmitoyl-2-oleoyl-*sn*- glycero-3-phosphocholine (POPC); 1, 2-dipalmitoyl-*sn*-phosphatidylcholine (DPPC) and 1, 2-d_62_-*sn*- dipalmitoyl-*sn*-phosphatidylcholine (DPPC-d_62_) were obtained from Avanti Polar Lipids (Birmingham, Alabama, USA). Idebenone was from TCI Europe N.W. (Zwijndrecht, Belgium) and all other chemicals were highly pure from Sigma Chemical Co. (Madrid, Spain).

## 3 Methods

### 3.1 Reduction of idebenone to idebenol

Idebenol was prepared from idebenone by reduction, using sodium borohydride, as described for the reduction of ubiquinone to ubiquinol [2].

### 3.2 X-ray diffraction

**Fig. 1:**
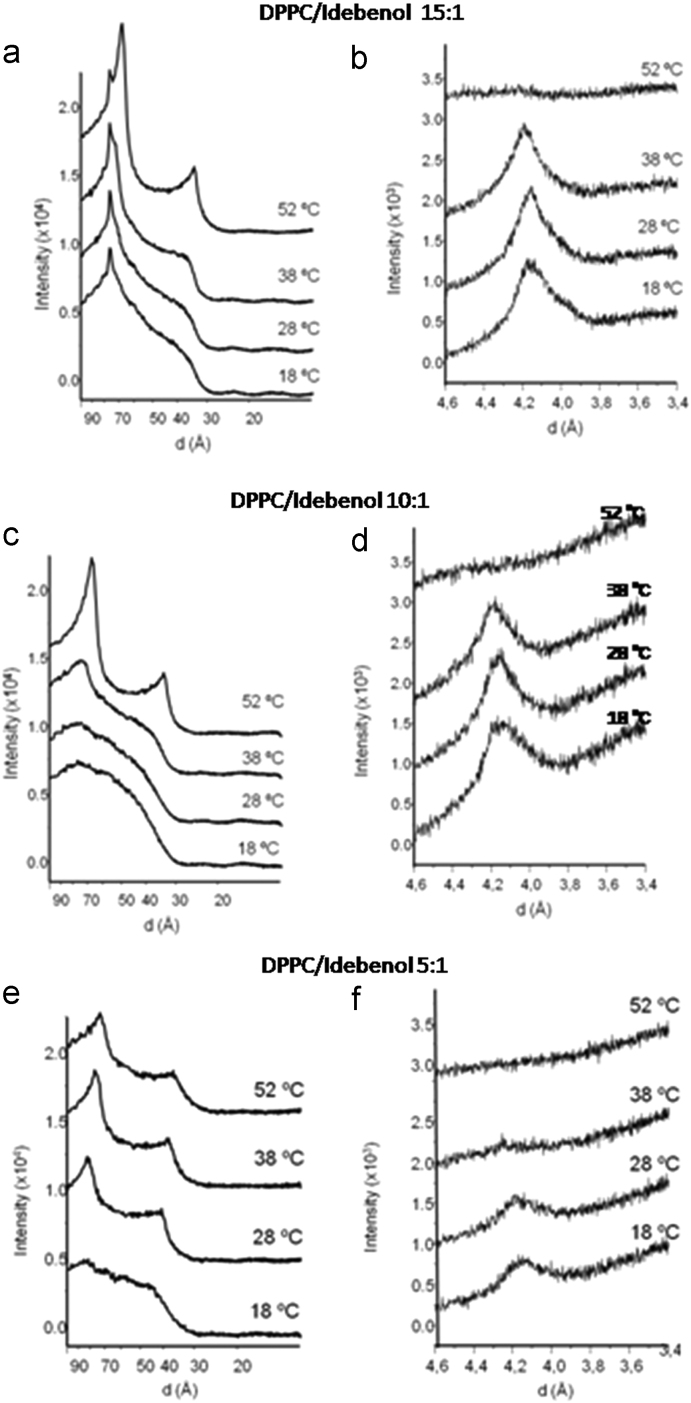
Small angle (A, C and E) and wide angle (B, D and F) diffraction profiles of DPPC/idebenol mixtures. Molar ratios and temperatures are shown next to the traces.

**Fig. 2:**
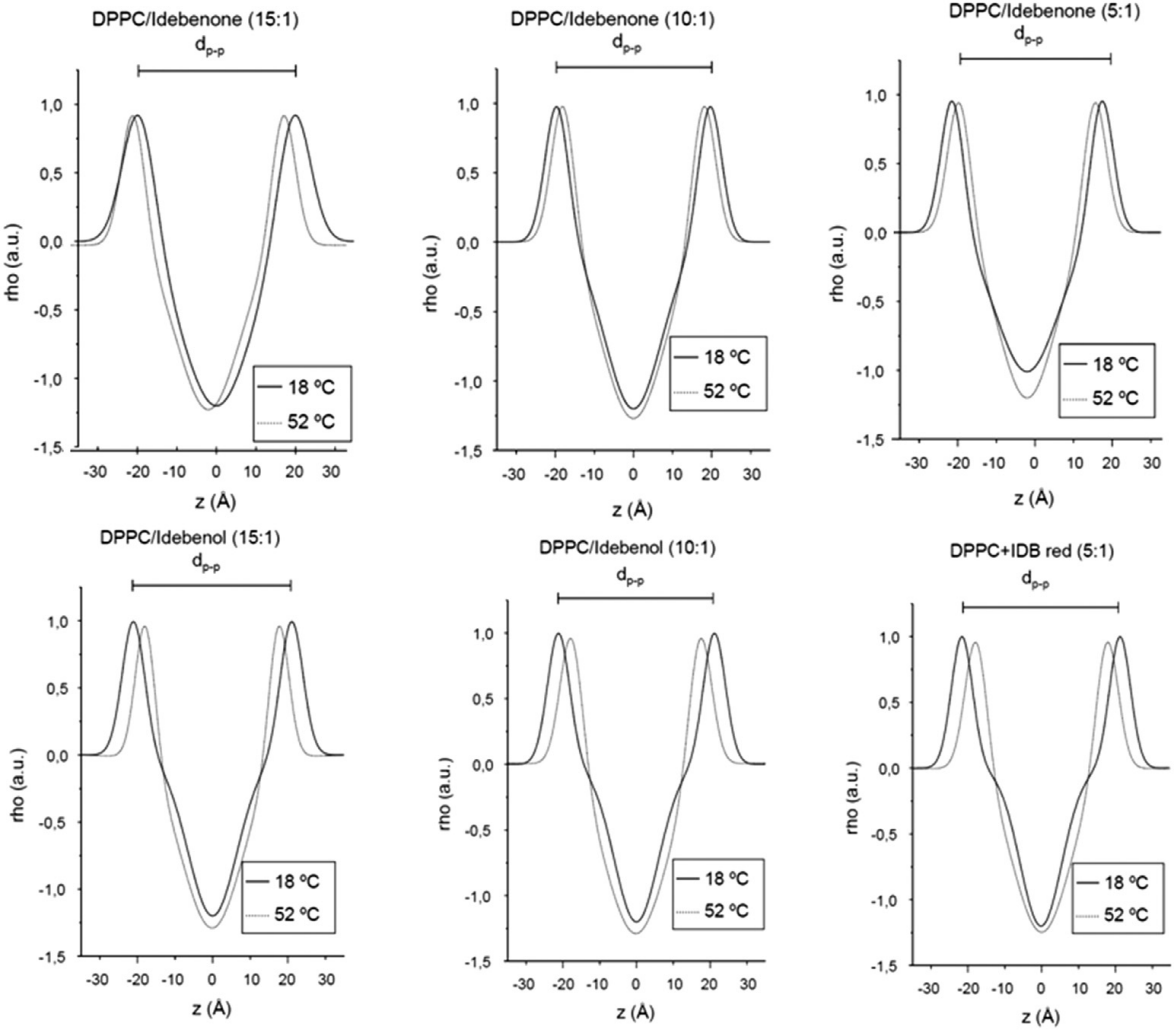
Small angle X-ray diffraction profiles at temperatures above (52 °C) and below(18 °C) of the main phase transition temperature of DPPC/idebenone and DPPC/idebenol mixtures. Molar ratios are indicated. Distances between the phosphate groups of bilayers (d_p-p_) are given in Å.

Samples for X-ray diffraction were prepared by dissolving 15 mg of DPPC/DPPCd_62_ (2:1 M ratio) and idebenone or idebenol to give the desired ratio, in in chloroform/methanol (2:1), evaporating the organic solvent under a stream of nitrogen and leaving the samples under vacuum for 3 hours. and the appropriate amount of idebenone or idebenol in chloroform/methanol (2:1). Multilamellar vesicles were formed by hydrating the samples in 0.5 mL of 100 mM NaCl, 25 mM Hepes pH 7.4 buffer by extensive vortexing, followed by centrifugation at 13,000*g*; the pellets were collected. Simultaneous small (SAXD) and wide (WAXD) angle X-ray diffraction measurements were carried out using a modified Kratky compact camera (MBraun-Graz-Optical Systems, Graz Austria), incorporating two coupled linear position sensitive detectors (PSD, MBraun, Garching, Germany) to monitor the s-ranges [s=2 sin *θ*/*λ*, 2*θ*=scattering angle, *λ*=1.54 Å] between 0.0075-0.07 and 0.20–0.29 Å^−1^, respectively. Nickel-filtered Cu KR X-rays were generated by a Philips PW3830 X-ray generator operating at 50 kV and 30 mA. The detector position was calibrated using Ag-stearate (small-angle region, D-spacing at 48.8 Å) and lupolen (wide angle region, D-spacing at 4.12 Å) as reference materials. Sample pellets were placed in a steel holder with cellophane windows, which provided good thermal contact with the Peltier heating unit. X-ray diffraction profiles were obtained for 10 min exposure times after 10 min of temperature equilibration. (Fig. 1; Table 1, Table 2) Background corrected SAXS data were analyzed using the program GAP (global analysis program) written by Georg Pabst and obtained from the author1 [3], [4]. This program allowed to retrieve the membrane thickness, from a full *q*-range analysis of the SAXS patterns [5]. The parameters *z*_H_ and *σ*_H_ are the position and width, respectively, of the Gaussian used to describe the electron-dense headgroup regions within the electron density model (Fig. 2).

### 3.3 ^2^H-NMR measurements

**Fig. 3:**
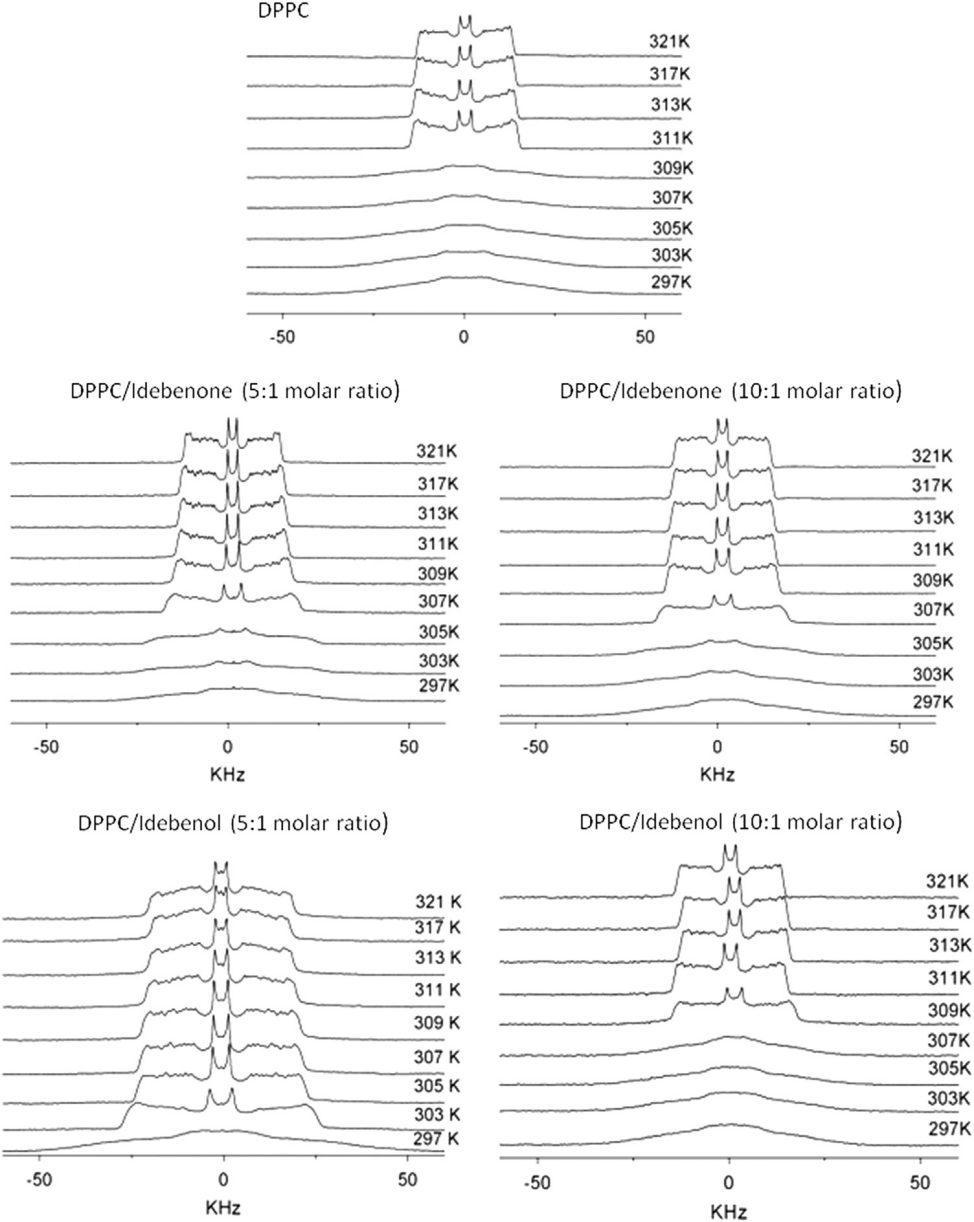
^2^H-NMR spectra of pure DPPC and DPPC/idebenone and DPPC/idebenol mixtures. Molar ratios and temperatures are indicated. In all the cases equimolar mixtures DPPC and DPPC-d_62_ are used.

Samples were prepared as described above for X-ray diffraction. Sample pellets were dispersed in 300 μl of buffer in deuterium-depleted water and transferred to NMR glass tubes. ^2^H-NMR experiments were carried out on a Bruker Avance 600 instrument (Bruker, Etlingen, Germany) at 92.123 MHz using the standard quadrupole echo sequence [6]. The spectral width was 150 KHz, with a 10 μs 90° pulse, 40 μs pulse spacing, 3.35 μs dwell time, 1 s recycling time and 50 Hz line broadening, with an accumulation of 15000 transients. Spectra were acquired at temperatures ranging from 18 °C to 52 °C, raising the temperature in 2 °C steps (Fig. 3).

Spectra were dePaked by numerical deconvolution with the software supplied by Bruker, Amix-tools versión 3.5.5, using the Thikonov regularization method. The dePaked spectra correspond to the spectra that would be obtained from a planar membrane with its bilayer normal aligned parallel to the applied static magnetic field, thus enhancing resolution and facilitating analysis of individual spectral peaks [6], [7], [8], [9]. These spectra were compared with the original ones to ensure that the relevant features were maintained during the dePakeing process.

### 3.4 ^31^P-NMR measurements

**Fig. 4:**
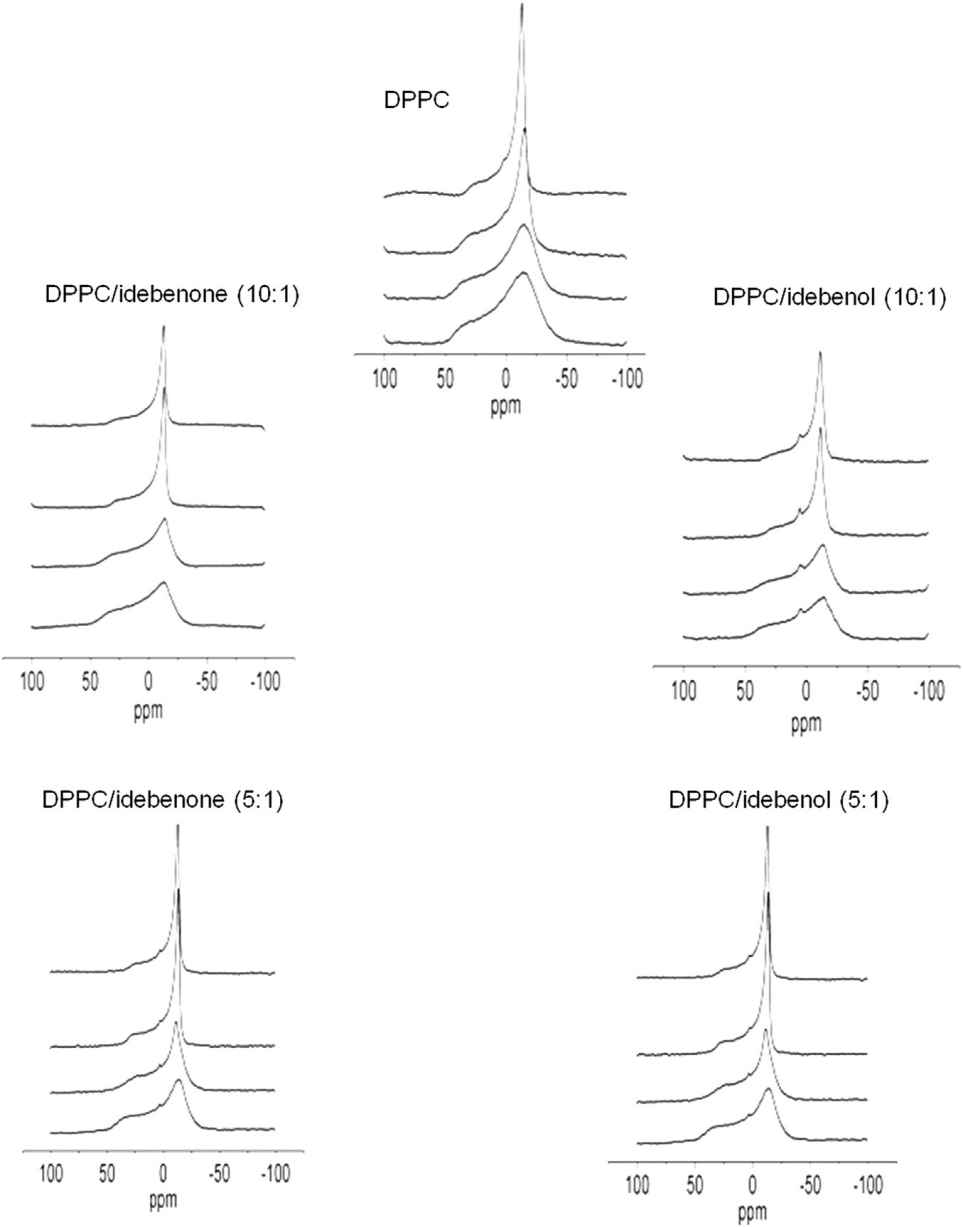
^31^P-NMR spectra of pure DPPC and mixtures DPPC/idebenone and DPPC/idebenol, at the molar ratios indicated. The spectra were obtained at different temperatures that are from bottom to top in all the cases: 18, 28, 38 and 52 °C.

The same samples and the same spectrometer operating at 242.9 MHz were also used to collect static ^31^P-NMR spectra. All spectra were obtained in the presence of a gated broad band proton decoupling (5 W input power during acquisition time), and accumulated free inductive decays were obtained from up to 8000 scans. A spectral width of 48,536 Hz, a memory of 48,536 data points, a 2 s interpulse time, and a 90° radio frequency pulse (11 μs) were used with inverse gated decoupling ^1^H. Prior to Fourier transformation, an exponential multiplication was applied, resulting in a 100 Hz line broadening (Fig. 4).

## Figures and Tables

**Table 1:** Repeat spacings (*d*, in nm) in the low-angle and wide angle regions of pure DPPC and DPPC/idebenone mixtures, at the molar ratios indicated, obtained after X-ray diffraction.

Mixture	Temperature (ºC)	Low-angle		Wide angle
		h, k	*d* (nm)	*d* (nm)
DPPC	52	1.0	67.29	
		2.0	33.66	
	38	1.0	74.90	4.18
		2.0	39.13	
	28	1.0	65.62	4.20–4.10
		2.0	32.70	
		3.0	21.73	
		4.0	16.34	
		5.0	13.15	
	18	1.0	65.08	4.20–4.10
		2.0	33.56	
		3.0	21.55	
		4.0	16.29	
		5.0	13.10	
				
DPPC/Idebenone 15:1)	52	1.0	75.62–68.45	
		2.0	34.24	
	38	1.0	75.62	4.18
		2.0	37.83	
	28	1.0	76.36	4.15
		2.0	42.70	
		3.0	24.09	
	18	1.0	76.34	4.14
		2.0	42.93	
		3.0	23.59	
				
DPPC/Idebenone (10:1)	52	1.0	75.62–67.86	
		2.0	34.10	
	38	1.0	75.62	4.19
		2.0	37.83	
	28	1.0	76.34	4.14
		2.0	42.70	
		3.0	24.24	
	18	1.0	76.34	4.14
		2.0	42.25	
		3.0	23.95	
				
DPPC/Idebenone (5:1)	52	1.0	75.62–69.65	
		2.0	35.00	
	38	1.0	74.90	
		2.0	37.29	
	28	1.0	80.20	4.15–3.92
		2.0	45.38	
		3.0	24.17	
	18	1.0	85.37	4.15
		2.0	46.72	
		3.0	24.24	

**Table 2:** Repeat spacings (*d*, in nm) in the low-angle and wide angle regions of DPPC/idebenol mixtures, at the molar ratios indicated, obtained after X-ray diffraction.

Mixture	Temperature (°C)	Low-angle		Wide angle
		h, k	*d* (nm)	*d* (nm)
DPPC/Idebenol (15:1)	52	1.0	76.36-67.86	
		2.0	34.10	
	38	1.0	76.34	4.18
		2.0	39.56	
	28	1.0	76.34	4.15
		2.0	41.80	
		3.0	23.32	
	18	1.0	76.34	4.16
		2.0	41.80	
		3.0	23.32	
DPPC/Idebenol (10:1)	52	1.0	67.29	
		2.0	33.71	
	38	1.0	74.20	4.19
		2.0	42.47	
		3.0	23.27	
		4.0	15.73	
	28	1.0	76.34	4.15
		2.0	23.27	
		3.0	15.73	
	18	1.0	77.08	4.15
		2.0	23.27	
		3.0	15.73	
DPPC/idebenol 5:1	52	1.0	73.52	
		2.0	36.61	
	38	1.0	77.08	
		2.0	38.56	
	28	1.0	82.70	4.15
		2.0	40.94	
	18	1.0	83.57	4.15
		2.0	43.88	
